# Variation of tRNA modifications with and without intron dependency

**DOI:** 10.3389/fgene.2024.1460902

**Published:** 2024-09-04

**Authors:** Sachiko Hayashi

**Affiliations:** Graduate School of Science, University of Hyogo, Ako-gun, Japan

**Keywords:** RNA modification, tRNA, intron, pre-tRNA, processing, enzyme

## Abstract

tRNAs have recently gained attention for their novel regulatory roles in translation and for their diverse functions beyond translation. One of the most remarkable aspects of tRNA biogenesis is the incorporation of various chemical modifications, ranging from simple base or ribose methylation to more complex hypermodifications such as formation of queuosine and wybutosine. Some tRNAs are transcribed as intron-containing pre-tRNAs. While the majority of these modifications occur independently of introns, some are catalyzed in an intron-inhibitory manner, and in certain cases, they occur in an intron-dependent manner. This review focuses on pre-tRNA modification, including intron-containing pre-tRNA, in both intron-inhibitory and intron-dependent fashions. Any perturbations in the modification and processing of tRNAs may lead to a range of diseases and disorders, highlighting the importance of understanding these mechanisms in molecular biology and medicine.

## 1 Introduction

**FIGURE 1:**
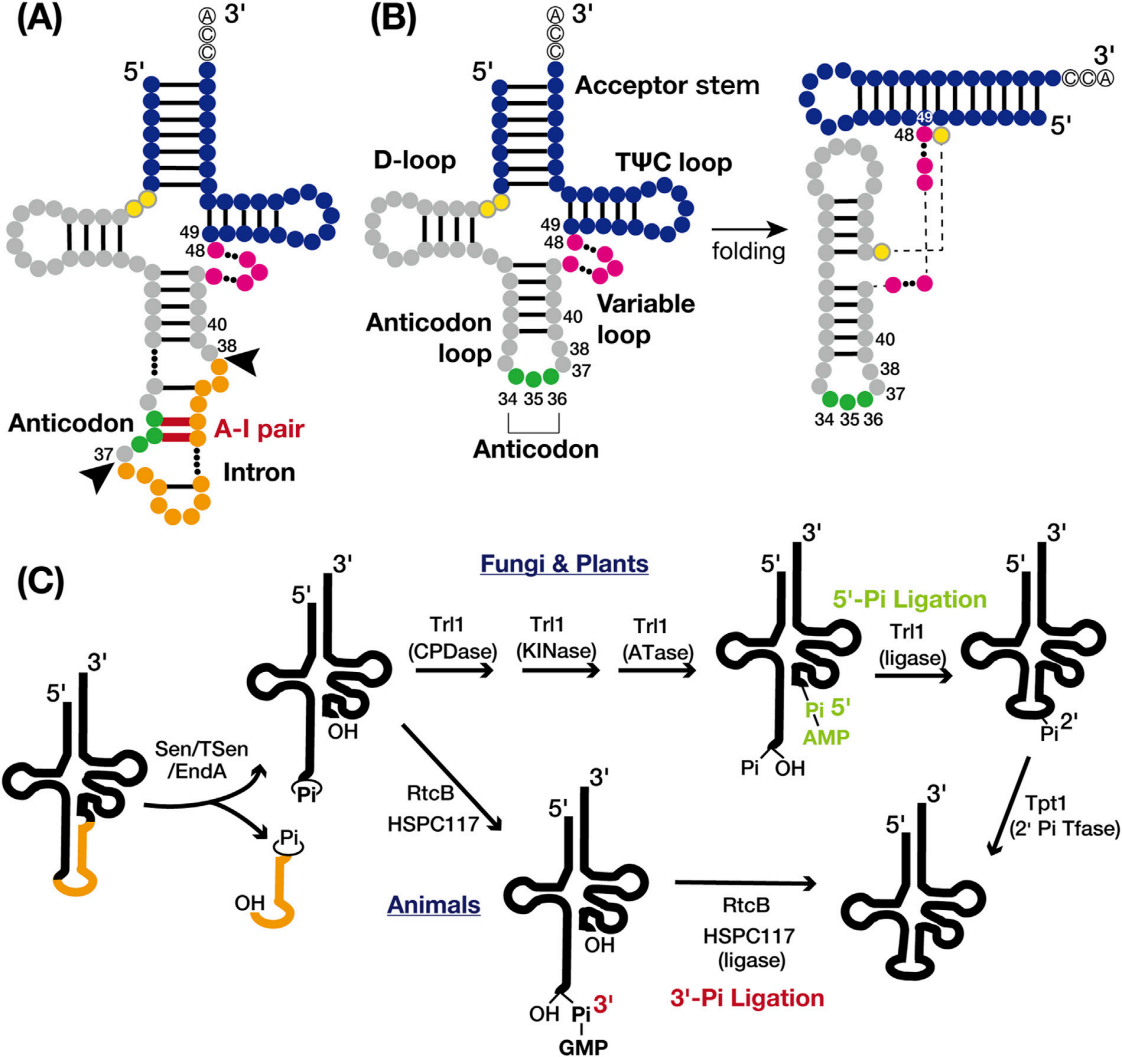
tRNA structures and tRNA splicing pathways. **(A)** Secondary structure of intron-containing pre-tRNA: the acceptor stem and TΨC loop (blue), D-loop and anticodon loop (gray), variable loop (pink), anticodon (green), and intron (orange). The intermediate nucleotides between the acceptor stem and D-loop are yellow with grey circles. Nucleotide base pairing is represented by black lines, with the anticodon-intron (A–I) pair highlighted in red. The nucleotide numbers indicate the positions adjacent to the intron insertion sites (positions 37/38) and the locations of known tRNA intron-dependent modification positions, excluding the anticodon (positions 34–36). Black arrows indicate the canonical intron insertion site. **(B)** Secondary (left) and tertiary (right) structures of mature tRNA are shown with the same color codes and nucleotide numbers as in A. **(C)** Cleavage of the tRNA splice site (left) and two distinct tRNA ligation pathways: the 5′-phosphate pathway (upper right) and the 3′-phosphate pathway (lower right). The tRNA body is represented in black and the intron in orange. Proteins involved in each chemical reaction are also shown.

Transfer RNAs (tRNAs) are small noncoding RNAs, typically 76 to 90 nucleotides long, forming a cloverleaf shape that folds into an L-shaped structure (Figures 1A, B). They undergo over 100 post-transcriptional modifications with 8–13 per molecule (Machnicka et al., 2014; Zhang et al., 2022; Cappannini et al., 2024). Modifications within the anticodon loop are essential for recognition by cognate aminoacyl-tRNA synthetases (aaRSs) and mRNA decoding accuracy, while modifications outside this region contribute to maintaining the structural integrity and quality control of tRNAs (Barraud and Tisné, 2019; Phizicky and Hopper, 2023). Recent advances in tRNA epitranscriptomics have mapped tRNA modifications and uncovered the roles of modification enzymes in various health and disease (Abbott et al., 2014; Kirchner and Ignatova, 2015; Ramos and Fu, 2019; Suzuki, 2021; Cerneckis et al., 2022; Sekulovski and Trowitzsch, 2022; Añazco-Guenkova et al., 2024; Liu et al., 2024). Comprehensive reviews further explore these discoveries, detailing the functions of newly identified tRNA modifications including the roles of tRNA fragments (Kessler et al., 2018b; Lyons et al., 2018; Schimmel, 2018; Krutyhołowa et al., 2019; George et al., 2022; Zhang et al., 2022; Phizicky and Hopper, 2023). This review explores the roles of tRNA introns in chemical modifications. In prokaryotes, tRNAs are often synthesized as multimeric precursors, processed to monomeric forms, trimmed 5′-3′, with CCA nucleotides at the 3′ end either encoded or added post-transcriptionally, and undergo nucleotide modifications (Mohanty and Kushner, 2019). In archaea and eukaryotes, tRNA processing starts with 5′-3′ trimming, followed by CCA addition, nucleotide modifications, and, for intron-containing pre-tRNAs, subsequent splicing of introns. The relationship between post-transcriptional modification and tRNA introns was pioneered and well-documented in the 1980s and 1990s (Johnson and Abelson, 1983; Strobel and Abelson, 1986; Choffat et al., 1988; Grosjean et al., 1997; Jiang et al., 1997; Motorin et al., 1998). However, the mechanisms involving intron-inhibitory, intron-dependent, and intron-insensitive enzymes proposed by (Grosjean et al., 1997) remain inadequately explained. Recent advancements in the field of modifications are likely to provide new insights into this complex and fascinating area of study, necessitating a revisitation of this topic.

## 2 tRNA intron and splicing

### 2.1 tRNA intron

tRNA introns were initially identified in yeast tRNA^Tyr^ genes by Goodman *et al.* in 1977 (Goodman et al., 1977) and are now widely recognized in both archaea and eukaryotes (Chan and Lowe, 2016). In bacteria, tRNA typically lacks introns spliced by specialized proteinaceous enzymes. Instead, the self-splicing group I introns, which use a mechanism related to mRNA splicing, are found in the anticodon region in cyanobacteria and some alpha- and beta-proteobacteria (Reinhold-Hurek and Shub, 1992; Tanner and Cech, 1996). In archaea, an estimated 15% of tRNA genes contain tRNA introns. Of those, approximately 75% are at the canonical 37/38 position, one nucleotide 3′ from the anticodon (Figure 1A). The remaining 25% are dispersed across various sites in tRNA genes, with some containing multiple introns predicted *in silico* (Marck and Grosjean, 2003; Sugahara et al., 2007; Tocchini-Valentini et al., 2009; 2011). In eukaryotes, 5%–25% of tRNA genes harbor introns, which are predominantly single and consistently at the canonical position 37/38 (Chan and Lowe, 2016; Hayne et al., 2023; Phizicky and Hopper, 2023; Zhang et al., 2023). These introns form an A-I pair that disrupts codon-anticodon binding during translation (Bufardeci et al., 1993), making tRNA splicing essential for pre-tRNA maturation (Figure 1A). The canonical position of tRNA introns is believed to be ancient (Fujishima and Kanai, 2014), suggesting that eukaryotes have retained tRNA introns over long evolutionary periods.

### 2.2 Conservation and diversity of tRNA splicing by proteins

tRNA splicing facilitated by specific endonucleases and ligases, involves key components which present across archaea to eukaryotes, albeit with variations in specific mechanisms. In archaea, tRNA splicing endonucleases and tRNA ligase process all introns, including those in pre-rRNAs and pre-mRNAs (Tocchini-Valentini et al., 2011; Tocchini-Valentini and Tocchini-Valentini, 2021). Most endonucleases recognize a structural motif called the bulge-helix-bulge (BHB) motif. In contrast, eukaryotes utilize a tRNA endonuclease (Sen/TSen) consisting of four conserved subunits, which cleaves splice sites via the “molecular ruler” mechanism (Trotta et al., 1997; Akama et al., 2000; Paushkin et al., 2004; Phizicky and Hopper, 2023). Two distinct ligation pathways exist for tRNA processing in both archaea and eukaryotes, categorized by the origin of the phosphate linking the 5′- and 3′-exons: the 5′-phosphate pathway and the 3′-phosphate pathway (Figure 1C). In eukaryotes, 5′-phosphate RNA ligases are common in fungi and plants, while 3′-phosphate RNA ligases are used in animals (Tocchini-Valentini et al., 2005; Popow et al., 2012; Yoshihisa, 2014; Phizicky and Hopper, 2023). The location of tRNA splicing varies among eukaryotes; in vertebrates and plants, tRNA splicing occurs within the nucleus, whereas in yeast, it occurs near the mitochondria due to different enzyme distributions (Melton et al., 1980; Yoshihisa et al., 2003; 2007; Paushkin et al., 2004; Mee et al., 2005). This spatial compartmentalization enables precise timing of chemical modifications during tRNA maturation, exhibiting variability across organisms and even within different tRNA genes of the same organism (Phizicky and Hopper, 2023). The rates of intracellular transport may influence the extent of modification (Kessler et al., 2018b). The complexity of this process is further amplified by the intricate nature of nucleoside modifications (Barraud and Tisné, 2019).

## 3 Modifications and tRNA introns

**FIGURE 2:**
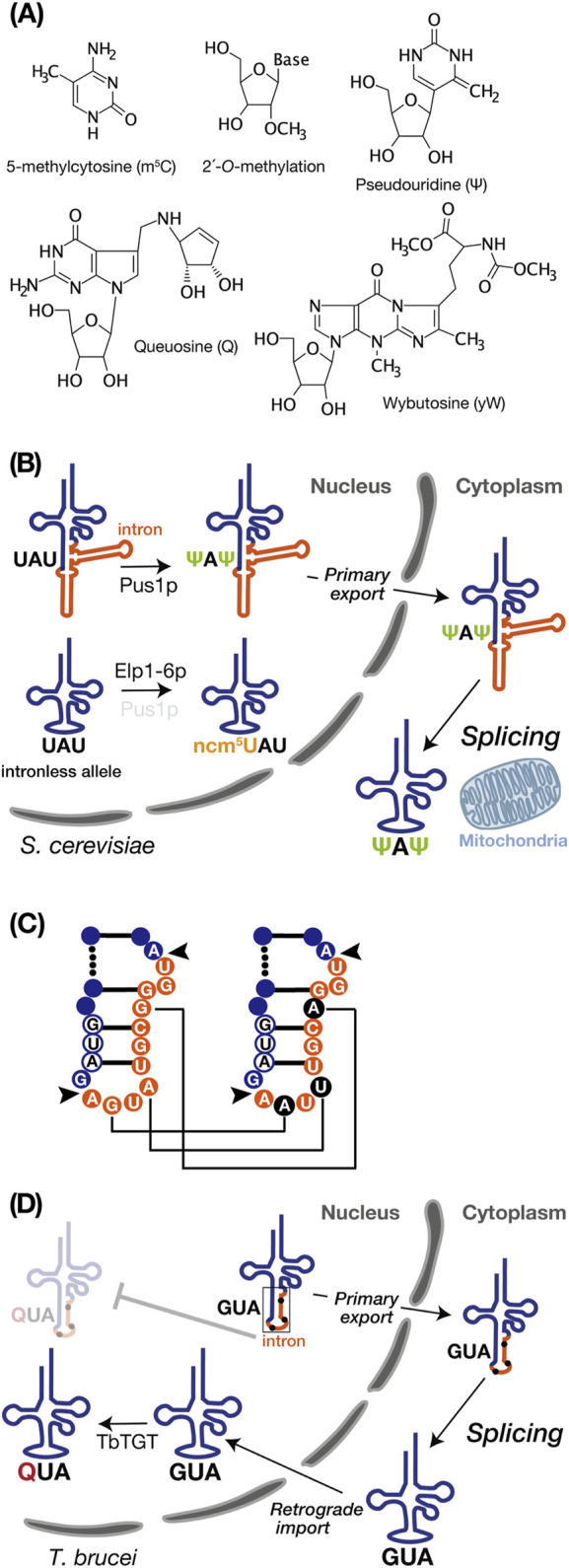
tRNA intron and modifications. **(A)** Chemical structures of the RNA modifications discussed in this review, created using MarvinSketch (ChemAxion Ltd.). **(B)** tRNA intron-dependent modification. In *S. cerevisiae*, pre-tRNA^Ile^_UAU_ undergoes intron-dependent pseudouridylation (Ψ) at U34 and U36 by Pus1p in the nucleus. Subsequently, the modified pre-tRNA is exported to the cytoplasm for splicing near the mitochondria. In the intronless allele, on the other hand, pre-tRNA^Ile^_UAU_ lacking an intron undergoes modification at position 34 by the Elp1-6p complex to convert ncm^5^U. **(C)** Schematic representation of non-canonical base editing of the intron in *Trypanosoma brucei* pre-tRNA^Tyr^_GUA_. The 11-nucleotide intron is shown in orange, and the GUA anticodon is depicted in white with a blue circle. Black arrows indicate the intron insertion sites. In the right panel, edited nucleotides are highlighted in black. Editing is essential for proper intron processing by the tRNA endonuclease (as shown in D). **(D)** In *Trypanosoma brucei*, the intron-containing pre-tRNA^Tyr^_GUA_ with edited intron, as shown on the right side of C, is exported to the cytoplasm as the primary export substrate. After cytoplasmic splicing, the post-spliced pre-tRNA^Tyr^_GUA_ undergoes intron-inhibitory modification with queuosine (Q) by *Trypanosoma brucei* tRNA-guanine transglycosylase (TbTGT).

Most of the diversity in tRNA modifications is concentrated within the anticodon loop, where these modifications are pivotal for accurately decoding mRNA at the ribosomal A-site (Machnicka et al., 2014). In intron-containing pre-tRNAs, the majority of modifications occur independently of introns, whether within or outside the anticodon-loop, some modifications are catalyzed only in an intron-dependent manner, and in specific cases, modifications occur in an intron-inhibitory manner. The following instances highlight these modifications (Figure 2A).

### 3.1 Pseudouridylation

Pseudouridylation of uridine (Ψ) is a common RNA modification catalyzed by pseudouridine synthase (Pus) enzymes across numerous noncoding and protein-coding RNA substrates (Rintala-Dempsey and Kothe, 2017; Purchal et al., 2022). Pseudouridine enables robust base pairing to A with stiffening the sugar-phosphate backbone and enhancing stacking interactions to adjacent base pairs, which yields genuine Watson–Crick-like base pairing as strong as a C-G pair (Davis, 1995; Spenkuch et al., 2014). In *Saccharomyces cerevisiae*, Pus1p pseudouridylates U34 and U36 in the anticodon of intron-containing pre-tRNA^Ile^_UAU_ but not its spliced form (Szweykowska-Kulinska et al., 1994; Simos et al., 1996; Motorin et al., 1998). *In vitro* biochemical studies revealed that removing either the acceptor stem, the TΨ loop, or the D stem-loop from the pre-tRNA did not affect the formation of Ψ_34_ and Ψ_36_, and even the anticodon stem-loop interrupted by the intron effectively serves as a substrate (Szweykowska-Kulinska et al., 1994). Structurally, Pus1p specifically recognizes the anticodon region only when the anticodon and the intron form a double helix (Motorin et al., 1998). This was also confirmed *in vivo* by systematic intron removal from the yeast tRNA genes (Hayashi et al., 2019). Interestingly, in tRNA^Ile^_UAU_ of the intronless mutant, some of U34 are aberrantly converted into 5-carbamoylmethyl U34 (ncm^5^U_34_). Notably, in the *pus1*Δ strain where intron-containing pre-tRNA^Ile^_UAU_ is transcribed, the ncm^5^U_34_ modification in tRNA^Ile^_UAU_ was also observed. Therefore, the intron in tRNA^Ile^_UAU_ serves as a positive factor for Ψ_34_ formation while concurrently preventing ncm^5^U_34_ formation (Figure 2B). Because the Elp complex (Elp1–6p), responsible for ncm^5^U_34_ formation in tRNAs, is localized in the nucleus (Huang et al., 2005; Huang et al., 2008) and does not recognize intron-containing pre-tRNA^Ile^_UAU_ (Yoshihisa et al., 2003), this modification in *pus1*Δ cells only occurs after splicing (Hayashi et al., 2019). Considering that the absence of Pus1p impairs the nuclear export of tRNAs, Pus1p-dependent tRNA modification seems to facilitate efficient nuclear export of certain tRNAs (Großhans et al., 2001). Thus, the intron-dependent Ψ_34_ modification or interaction with Pus1p may play some roles in pre-tRNA^Ile^_UAU_ export for tRNA splicing near mitochondria.

Another example of intron-dependent pseudouridylation is Ψ_35_ introduction to tRNA^Tyr^_GUA_ by Pus7p (van Tol and Beier, 1988; Motorin et al., 1998; Behm-Ansmant et al., 2003; Urban et al., 2009). Pus7p incorporates pseudouridines into a particularly diverse set of RNAs, including tRNA, small nuclear RNA (snRNA), rRNA, and mRNA (Behm-Ansmant et al., 2003; Carlile et al., 2014; Schwartz et al., 2014; Guzzi et al., 2018). The presence of Ψ_35_ in tRNA is determined by the nucleotide sequence around U35 and is affected by the length of the intron rather than its specific structural characteristics (Szweykowska-Kulinska and Beier, 1992). tRNA^Tyr^_GUA_ with Ψ35 often acts as a suppressor for amber (UAG) and ochre (UAA) stop codons (Johnson and Abelson, 1983; Zerfass and Beier, 1992). The absence of Ψ35 significantly reduces its near-cognate suppressor activity at these stop codons but does not impact the decoding of cognate tyrosine codons (Johnson and Abelson, 1983; Blanchet et al., 2018). Additionally, the combination of Ψ_35_ and intron-independent A37 isopentenylation (i^6^A_37_) by Mod5p is crucial for effective stop codon suppression (Blanchet et al., 2018). While Ψ_35_ primarily aids in recognizing the UAG codon, i^6^A_37_ is more influential in readthrough of the UAA stop codon. Importantly, Ψ_35_-containing tRNA^Tyr^_GUA_ does not distinguish between the two synonymous tyrosine codons, UAU and UAC (Blanchet et al., 2018). Thus, the existence of tRNA^Tyr^_GUA_ introns seems to be meaningful for amber/ochre suppression. Considering that nonsense mutations, associated with nearly 1,000 serious genetic disorders, account for 10%–15% of all genetic defects that lead to disease (Mort et al., 2008), the role of suppressor tRNA^Tyr^ in amber/ochre suppression may have significant contribute to preventing and treating these diseases.

### 3.2 Base methylation

Methylations of tRNA molecules influence various aspects such as their folding dynamics, stability under heat, maturation process, and protection against cleavage, while also priming them for the synthesis of subsequent modifications (Hori, 2014; Zhang et al., 2022). Eukaryotic tRNA methyltransferases (TRMs) commonly employ S-adenosyl methionine (SAM) as a methyl donor (Lesnik et al., 2015). The formation of 5-methylcytosine at position 34 (m^5^C34) of tRNA^Leu^_CAA_ requires its intron and occurs in the nucleus both in *S. cerevisiae* and in human (Motorin and Grosjean, 1999; Auxilien et al., 2012). The structure of a prolonged anticodon stem and the nucleotide sequence surrounding the position to be modified are crucial for m^5^C34 formation in pre-tRNA^Leu^_CAA_ (Brzezicha et al., 2006). The wobble base modification of tRNA^Leu^_CAA_ is implicated in regulating translation. In yeast, under oxidative stress conditions, there is an upregulation of the m^5^C34 modification, which enhances translation of the corresponding Leu (UUG) codons (Chan et al., 2012). In human, m^5^C34 is partially hydroxylated to form 5-hydroxymethyl-2′-O-methylcytidine (hm^5^C34), which then undergoes further oxidation to yield 5-formylcytidine (f^5^C34), and their 2′-*O*-methylation (f^5^Cm34/hm^5^Cm34) (Kawarada et al., 2017). Pathogenic mutations in FTSJ1 and TRM4/NSUN2, involving in the process, are associated with intellectual disabilities. Dysregulation of protein synthesis due to the loss of f^5^Cm34/hm^5^Cm34 in cytoplasmic tRNA^Leu^_CAA_ may contribute to the molecular pathogenesis of these diseases (Kawarada et al., 2017). Additionally, in yeast pre-tRNA^Phe^_GAA_, an intron-dependent methylation by Trm4p occurs outside the anticodon, but in the anticodon stem-loop, at position C40 (Jiang et al., 1997; Motorin and Grosjean, 1999). This m^5^C40 modification is crucial for the spatial organization of the anticodon stem-loop and the formation of the Mg^2+^ binding pocket (Agris, 1996).

In contrast, in *S. cerevisiae*, Trm4p methylates on C48 and C49 in an intron-independent manner. Typically, this modification occurs at either C48 or C49 alone, but not simultaneously at both sites (Grosjean et al., 1997; Motorin et al., 2009; Wang et al., 2023). This pattern of methylation is conserved in humans, where intron-independent m^5^C48 formation on tRNA^Leu^_CAA_ is also observed (Auxilien et al., 2012). Thus, m^5^C40 of yeast tRNA^Phe^_GAA_ and m^5^C34 of yeast and human tRNA^Leu^_CAA_ are introduced intron-dependently, while m^5^C49 of yeast tRNA^Phe^_GAA_ and m^5^C48 of human tRNA^Leu^_CAA_ are intron-independently. Consequently, Trm4p introduces modifications within the same molecules using different recognition mechanisms.

In a fission yeast *S. pombe* and a dicotyledon *Arabidopsis thaliana*, as well as some other plants, there are two homologs of Trm4p/NSun2 termed Trm4a and Trm4b (Becker et al., 2012; David et al., 2017). *Schizosaccharomyces pombe* methylases exhibit a clear division of labor *in vivo*: Trm4a methylates all C48 residues and also C34 in tRNA^Leu^_CAA_ and tRNA^Pro^_CGG_, whereas Trm4b specifically methylates C49 (Müller et al., 2019). *In vitro*, however, Trm4b acts on C34 intron-dependently though inefficiently (Müller et al., 2019), suggesting that such Trm4b activity on C34 is somehow completely suppressed *in vivo*.

### 3.3 Ribose methylation: 2′-*O*-methylation

In a certain case, tRNA introns act as guiding elements that designate specific nucleotide positions for modifications. In archaea and eukaryotes, most 2′-*O*-methylations in ribosomal and other RNAs are directed by box C/D and box H/ACA small nucleolar RNPs (snoRNPs), respectively (Watkins and Bohnsack, 2012; Kufel and Grzechnik, 2019; Breuer et al., 2021). Archaea present an additional scenario where a functional unit for tRNA modification, such as a box C/D small RNA, is embedded within the tRNA’s intron, as seen in the tRNA^Trp^ precursor of *Haloferax volcanii* and other Euryarchaeota (D’Orval et al., 2001). This intronic segment, or the excised intron, facilitates 2′-*O*-methylation of C34 and U39 *in trans* (Singh et al., 2004). Deleting the box C/D RNA-containing intron abolishes RNA-guided 2′-*O* methylations of C34 and U39 without affecting growth under standard conditions (Joardar et al., 2008).

### 3.4 Queuosine

Queuosine (Q) is a hypermodified nucleotide derived from guanosine, incorporated at the wobble anticodon position 34 of tRNAs bearing the 5′-GUN-3′ (N = any base) anticodon sequence, which decode codons for Asn, Asp, His, and Tyr (Fergus et al., 2015). Q is present in both bacteria and eukaryotes, but it is only synthesized *de novo* in bacteria. Therefore, eukaryotes entirely rely Q supply on external sources originated from bacteria (Fergus et al., 2015). In *T. brucei*, only the cytoplasmically spliced pre-tRNA^Tyr^_GUA_, transported back to the nucleus via retrograde transport pathway, can undergoes Q modification facilitated by tRNA-guanine transglycosylase which localized in the nucleus (Kessler et al., 2018a) (Figure 2D). Q-modified tRNA^Tyr^_GUA_ is subsequently re-exported to the cytoplasm for translation, and a portion is selectively imported into mitochondria (Hegedűsová et al., 2019; Kulkarni et al., 2021). Due to varying steady-state levels of Q among different life cycle stages of *Trypanosoma brucei*, this modification has significant roles in codon selection in these parasite stages (Dixit et al., 2021). Interestingly, these sequential processing events begin with non-canonical editing, including guanosine-to-adenosine transitions (G to A) and an adenosine-to-uridine transversion (A to U), on the intron of pre-tRNA^Tyr^_GUA_, which is essential for maturation because recognition and splicing by the SEN complex occur exclusively with the edited form of pre-tRNA^Tyr^_GUA_ (Rubio et al., 2013) (Figure 2C). So far, mechanisms behind these non-canonical intron editing are ambiguous, but they appear to involve neither enzymatic activity nor deamination. Instead, they are likely related to nucleotide or base replacement (Rubio et al., 2013). Collectively, the *T. brucei* tRNA^Tyr^_GUA_ intron negatively governs Q modification as a barrier, and splicing of this intron is under the control of nucleotide editing.

### 3.5 Wybutosine

Wybutosine base (yW) is a complex nucleotide on tRNAs originating from a genetically encoded guanosine residue, undergoing a transformation into a fluorescent tricyclic fused aromatic base (Noma et al., 2006; Perche-Letuvée et al., 2014). Typically found at position 37 of eukaryotic and archaeal tRNA^Phe^_GAA_, yW and its derivatives stabilize the first base pair of the codon-anticodon duplex in the ribosomal A site through base stacking (Konevega et al., 2004). Although yW does not significantly affect the aminoacylation of tRNA^Phe^_GAA_ (Thiebe and Zachau, 1968), its absence promotes frameshifting and impacts viral RNA replication (Waas et al., 2007). Similarly to Q modification in *T. brucei* tRNA^Tyr^_GUA_, only spliced pre-tRNA^Phe^_GAA_ in *S. cerevisiae* undergoes yW modification in *S. cerevisiae* (Ohira and Suzuki, 2011). After splicing, tRNA^Phe^_GAA_ is re-imported into the nucleus where Trm5p catalyzes the formation of m^1^G37, the first step of yW formation. The resulting tRNA^Tyr^ intermediate is then exported back to the cytoplasm, where the remaining enzymes such as Tyw1p, Tyw2p, Tyw3p, and Tyw4p complete yW synthesis. Thus, tRNA^Phe^_GAA_ intron regulates proper timing of yW modification steps during nuclear-cytoplasmic shuttling of tRNA^Phe^_GAA_. Structural analysis of *Pyrococcus abyssi* Trm5a reveals that the enzyme recognizes the overall shape of tRNA (Wang et al., 2017), suggesting that the intron-containing pre-tRNA^Phe^_GAA_, with its disrupted anticodon-loop structure due to the A-I pair, could escape from recognition by Trm5p before splicing.

## 4 Conclusion

A number of tRNA modifications, especially those located outside the anticodon region, occur independently of the presence or absence of intron (Grosjean et al., 1997). A study of yeast intronless alleles revealed that none of the strains displayed severe growth defects under normal conditions, and the majority maintained relatively mature tRNA expression, aminoacylation status, and general translation capacity (Hayashi et al., 2019). Thus, tRNA introns may have a limited impact on tRNA biology, including nucleotide modifications. However, certain modification enzymes selectively recognize targets and modify specific sites within the same tRNA molecules through either an intron-dependent or -independent mechanism. Unlike mRNA introns, which lead to the diversity of mature mRNA sequences through splicing, tRNA introns do not contribute to tRNA primary sequence diversity. Since RNA modifications provide an additional layer of regulation beyond the primary sequence, organisms may have evolved intron-dependent or intron-inhibitory modifications to delicately adjust tRNA quantity and/or function by controlling the order and timing of certain modifications during tRNA maturation. In a different view, intron-insensitive tRNA modifications might serve more fundamental and essential roles on tRNA. The precise mechanism behind this selectivity involving introns remains incompletely understood. Further investigation into regulatory roles of introns in these modifications holds significant potential for discovery. How can individual modifications be distinguished as intron-dependent or -independent? What explains the conservation of intron-dependent modifications in specific tRNA molecules across different species, despite variations in their tRNA intron sequences? How did modification enzymes evolve to accommodate changes in intron sequences? Addressing these questions would not only enhance our comprehension of fundamental molecular mechanisms of tRNA maturation but also advance our understanding of evolutionary adaptations of this complicated process. Additionally, these insights may inform developments in treatments and drugs targeting tRNA modifications in diseases.

## References

[B1] Abbott J. A. Francklyn C. S. Robey-Bond S. M. (2014). Transfer RNA and human disease. Front. Genet. 5, 158. 10.3389/fgene.2014.00158 24917879 PMC4042891

[B2] Agris P. F. (1996). The importance of being modified: roles of modified nucleosides and Mg2^+^ in RNA structure and function. Prog. Nucleic Acid. Res. Mol. Biol. 53, 79–129. 10.1016/S0079-6603(08)60143-9 8650309

[B3] Akama K. Junker V. Beier H. (2000). Identification of two catalytic subunits of tRNA splicing endonuclease from *Arabidopsis thaliana* . Gene 257, 177–185. 10.1016/S0378-1119(00)00408-X 11080584

[B4] Añazco-Guenkova A. M. Miguel-López B. Monteagudo-García Ó. García-Vílchez R. Blanco S. (2024). The impact of tRNA modifications on translation in cancer: identifying novel therapeutic avenues. Nar. Cancer 6, zcae012. 10.1093/narcan/zcae012 38476632 PMC10928989

[B5] Auxilien S. Guérineau V. Szweykowska-Kuliñska Z. Golinelli-Pimpaneau B. (2012). The human tRNA m^5^C methyltransferase Misu is multisite-specific. RNA Biol. 9, 1331–1338. 10.4161/rna.22180 22995836 PMC3597573

[B6] Barraud P. Tisné C. (2019). To be or not to be modified: miscellaneous aspects influencing nucleotide modifications in tRNAs. IUBMB Life 71, 1126–1140. 10.1002/iub.2041 30932315 PMC6850298

[B7] Becker M. Müller S. Nellen W. Jurkowski T. P. Jeltsch A. Ehrenhofer-Murray A. E. (2012). Pmt1, a Dnmt2 homolog in *Schizosaccharomyces pombe*, mediates tRNA methylation in response to nutrient signaling. Nucleic Acids Res. 40, 11648–11658. 10.1093/nar/gks956 23074192 PMC3526270

[B8] Behm-Ansmant I. Urban A. Ma X. Yu Y.-T. Motorin Y. Branlant C. (2003). The *Saccharomyces cerevisiae* U2 snRNA:pseudouridine-synthase Pus7p is a novel multisite–multisubstrate RNA:Ψ-synthase also acting on tRNAs. RNA 9, 1371–1382. 10.1261/rna.5520403 14561887 PMC1287059

[B9] Blanchet S. Cornu D. Hatin I. Grosjean H. Bertin P. Namy O. (2018). Deciphering the reading of the genetic code by near-cognate tRNA. Proc. Natl. Acad. Sci. U. S. A. 115, 3018–3023. 10.1073/pnas.1715578115 29507244 PMC5866558

[B10] Breuer R. Gomes-Filho J. V. Randau L. (2021). Conservation of archaeal C/D box sRNA-guided RNA modifications. Front. Microbiol. 12, 654029. 10.3389/fmicb.2021.654029 33776983 PMC7994747

[B11] Brzezicha B. Schmidt M. Makałowska I. Jarmołowski A. Pieńkowska J. Szweykowska-Kulińska Z. (2006). Identification of human tRNA: m^5^C methyltransferase catalysing intron-dependent m^5^C formation in the first position of the anticodon of the pre-tRNA^Leu^ _(CAA)_ . Nucleic Acids Res. 34, 6034–6043. 10.1093/nar/gkl765 17071714 PMC1635329

[B12] Bufardeci E. Fabbri S. Baldi M. I. Mattoccia E. Tocchini-Valentini G. P. (1993). *In vitro* genetic analysis of the structural features of the pre-tRNA required for determination of the 3′ splice site in the intron excision reaction. EMBO J. 12, 4697–4704. 10.1002/j.1460-2075.1993.tb06158.x 8223479 PMC413913

[B13] Cappannini A. Ray A. Purta E. Mukherjee S. Boccaletto P. Moafinejad S. N. (2024). MODOMICS: a database of RNA modifications and related information. 2023 update. Nucleic Acids Res. 52, D239–D244. 10.1093/nar/gkad1083 38015436 PMC10767930

[B14] Carlile T. M. Rojas-Duran M. F. Zinshteyn B. Shin H. Bartoli K. M. Gilbert W. V. (2014). Pseudouridine profiling reveals regulated mRNA pseudouridylation in yeast and human cells. Nature 515, 143–146. 10.1038/nature13802 25192136 PMC4224642

[B15] Cerneckis J. Cui Q. He C. Yi C. Shi Y. (2022). Decoding pseudouridine: an emerging target for therapeutic development. Trends Pharmacol. Sci. 43, 522–535. 10.1016/j.tips.2022.03.008 35461717

[B16] Chan C. T. Y. Pang Y. L. J. Deng W. Babu I. R. Dyavaiah M. Begley T. J. (2012). Reprogramming of tRNA modifications controls the oxidative stress response by codon-biased translation of proteins. Nat. Commun. 3, 937. 10.1038/ncomms1938 22760636 PMC3535174

[B17] Chan P. P. Lowe T. M. (2016). GtRNAdb 2.0: an expanded database of transfer RNA genes identified in complete and draft genomes. Nucleic Acids Res. 44, D184–D189. 10.1093/nar/gkv1309 26673694 PMC4702915

[B18] Choffat Y. Suter B. Behra R. Kubli E. (1988). Pseudouridine modification in the tRNA(Tyr) anticodon is dependent on the presence, but independent of the size and sequence, of the intron in eucaryotic tRNA(Tyr) genes. Mol. Cell. Biol. 8, 3332–3337. 10.1128/mcb.8.8.3332 3145410 PMC363568

[B19] David R. Burgess A. Parker B. Li J. Pulsford K. Sibbritt T. (2017). Transcriptome-wide mapping of RNA 5-methylcytosine in arabidopsis mRNAs and noncoding RNAs. Plant Cell 29, 445–460. 10.1105/tpc.16.00751 28062751 PMC5385953

[B20] Davis D. R. (1995). Stabilization of RNA stacking by pseudouridine. Nucleic Acids Res. 23, 5020–5026. 10.1093/nar/23.24.5020 8559660 PMC307508

[B21] Dixit S. Kessler A. C. Henderson J. Pan X. Zhao R. D’Almeida G. S. (2021). Dynamic queuosine changes in tRNA couple nutrient levels to codon choice in *Trypanosoma brucei* . Nucleic Acids Res. 49, 12986–12999. 10.1093/nar/gkab1204 34883512 PMC8682783

[B22] D’Orval B. C. Bortolin M. L. Gaspin C. Bachellerie J. P. (2001). Box C/D RNA guides for the ribose methylation of archaeal tRNAs. The tRNATrp intron guides the formation of two ribose-methylated nucleosides in the mature tRNATrp. Nucleic Acids Res. 29, 4518–4529. 10.1093/nar/29.22.4518 11713301 PMC92551

[B23] Fergus C. Barnes D. Alqasem M. Kelly V. (2015). The queuine micronutrient: charting a course from microbe to man. Nutrients 7, 2897–2929. 10.3390/nu7042897 25884661 PMC4425180

[B24] Fujishima K. Kanai A. (2014). tRNA gene diversity in the three domains of life. Front. Genet. 5, 142. 10.3389/fgene.2014.00142 24904642 PMC4033280

[B25] George S. Rafi M. Aldarmaki M. ElSiddig M. Al Nuaimi M. Amiri K. M. A. (2022). tRNA derived small RNAs—small players with big roles. Front. Genet. 13, 997780. 10.3389/fgene.2022.997780 36199575 PMC9527309

[B26] Goodman H. M. Olson M. V. Hall B. D. (1977). Nucleotide sequence of a mutant eukaryotic gene: the yeast tyrosine-inserting ochre suppressor SUP4-o. Proc. Natl. Acad. Sci. U. S. A. 74, 5453–5457. 10.1073/pnas.74.12.5453 341157 PMC431761

[B27] Grosjean H. Szweykowska-Kulinska Z. Motorin Y. Fasiolo F. Simos G. (1997). Intron-dependent enzymatic formation of modified nucleosides in eukaryotic tRNAs: a review. Biochimie 79, 293–302. 10.1016/S0300-9084(97)83517-1 9258438

[B28] Großhans H. Lecointe F. Grosjean H. Hurt E. Simos G. (2001). Pus1p-dependent tRNA pseudouridinylation becomes essential when tRNA biogenesis is compromised in yeast. J. Biol. Chem. 276, 46333–46339. 10.1074/jbc.M107141200 11571299

[B29] Guzzi N. Cieśla M. Ngoc P. C. T. Lang S. Arora S. Dimitriou M. (2018). Pseudouridylation of tRNA-derived fragments steers translational control in stem cells. Cell 173, 1204–1216. 10.1016/j.cell.2018.03.008 29628141

[B30] Hayashi S. Mori S. Suzuki T. Suzuki T. Yoshihisa T. (2019). Impact of intron removal from tRNA genes on *Saccharomyces cerevisiae* . Nucleic Acids Res. 47, 5936–5949. 10.1093/nar/gkz270 30997502 PMC6582322

[B31] Hayne C. K. Butay K. J. U. Stewart Z. D. Krahn J. M. Perera L. Williams J. G. (2023). Structural basis for pre-tRNA recognition and processing by the human tRNA splicing endonuclease complex. Nat. Struct. Mol. Biol. 30, 824–833. 10.1038/s41594-023-00991-z 37231153 PMC10627149

[B32] Hegedűsová E. Kulkarni S. Burgman B. Alfonzo J. D. Paris Z. (2019). The general mRNA exporters Mex67 and Mtr2 play distinct roles in nuclear export of tRNAs in *Trypanosoma brucei* . Nucleic Acids Res. 47, 8620–8631. 10.1093/NAR/GKZ671 31392978 PMC6794378

[B33] Hori H. (2014). Methylated nucleosides in tRNA and tRNA methyltransferases. Front. Genet. 5, 144. 10.3389/fgene.2014.00144 24904644 PMC4033218

[B34] Huang B. Jian L. Byström A. S. (2008). A genome-wide screen identifies genes required for formation of the wobble nucleoside 5-methoxycarbonylmethyl-2-thiouridine in *Saccharomyces cerevisiae* . RNA 14, 2183–2194. 10.1261/rna.1184108 18755837 PMC2553728

[B35] Huang B. Johansson M. J. O. Byström A. S. (2005). An early step in wobble uridine tRNA modification requires the Elongator complex. RNA 11, 424–436. 10.1261/rna.7247705 15769872 PMC1370732

[B36] Jiang H. Q. Motorin Y. Jin Y. X. Grosjean H. (1997). Pleiotropic effects of intron removal on base modification pattern of yeast tRNA^Phe^: an *in vitro* study. Nucleic Acids Res. 25, 2694–2701. 10.1093/nar/25.14.2694 9207014 PMC146816

[B37] Joardar A. Gurha P. Skariah G. Gupta R. (2008). Box C/D RNA-guided 2′-O methylations and the intron of tRNA^Trp^ are not essential for the viability of *Haloferax volcanii* . J. Bacteriol. 190, 7308–7313. 10.1128/JB.00820-08 18757532 PMC2580716

[B38] Johnson P. F. Abelson J. (1983). The yeast tRNA^Tyr^ gene intron is essential for correct modification of its tRNA product. Nature 302, 681–687. 10.1038/302681a0 6339954

[B39] Kawarada L. Suzuki T. Ohira T. Hirata S. Miyauchi K. Suzuki T. (2017). ALKBH1 is an RNA dioxygenase responsible for cytoplasmic and mitochondrial tRNA modifications. Nucleic Acids Res. 45, 7401–7415. 10.1093/nar/gkx354 28472312 PMC5499545

[B40] Kessler A. C. Kulkarni S. S. Paulines M. J. Rubio M. A. T. Limbach P. A. Paris Z. (2018a). Retrograde nuclear transport from the cytoplasm is required for tRNA^Tyr^ maturation in *T. brucei* . RNA Biol. 15, 528–536. 10.1080/15476286.2017.1377878 28901827 PMC6103694

[B41] Kessler A. C. Silveira d’Almeida G. Alfonzo J. D. (2018b). The role of intracellular compartmentalization on tRNA processing and modification. RNA Biol. 15, 554–566. 10.1080/15476286.2017.1371402 28850002 PMC6103688

[B42] Kirchner S. Ignatova Z. (2015). Emerging roles of tRNA in adaptive translation, signalling dynamics and disease. Nat. Rev. Genet. 16, 98–112. 10.1038/nrg3861 25534324

[B43] Konevega A. L. Soboleva N. G. Makhno V. I. Semenkov Y. P. Wintermeyer W. Rodnina M. V. (2004). Purine bases at position 37 of tRNA stabilize codon-anticodon interaction in the ribosomal A site by stacking and Mg^2+^-dependent interactions. RNA 10, 90–101. 10.1261/rna.5142404 14681588 PMC1370521

[B44] Krutyhołowa R. Zakrzewski K. Glatt S. (2019). Charging the code — tRNA modification complexes. Curr. Opin. Struct. Biol. 55, 138–146. 10.1016/j.sbi.2019.03.014 31102979

[B45] Kufel J. Grzechnik P. (2019). Small nucleolar RNAs tell a different tale. Trends Genet. 35, 104–117. 10.1016/j.tig.2018.11.005 30563726

[B46] Kulkarni S. Rubio M. A. T. Hegedusová E. Ross R. L. Limbach P. A. Alfonzo J. D. (2021). Preferential import of queuosine-modified tRNAs into *Trypanosoma brucei* mitochondrion is critical for organellar protein synthesis. Nucleic Acids Res. 49, 8247–8260. 10.1093/nar/gkab567 34244755 PMC8373054

[B47] Lesnik C. Golani-Armon A. Arava Y. (2015). Localized translation near the mitochondrial outer membrane: an update. RNA Biol. 12, 801–809. 10.1080/15476286.2015.1058686 26151724 PMC4615199

[B48] Liu W. W. Zheng S. Q. Li T. Fei Y. F. Wang C. Zhang S. (2024). RNA modifications in cellular metabolism: implications for metabolism-targeted therapy and immunotherapy. Signal Transduct. Target. Ther. 9, 70. 10.1038/s41392-024-01777-5 38531882 PMC10966055

[B49] Lyons S. M. Fay M. M. Ivanov P. (2018). The role of RNA modifications in the regulation of tRNA cleavage. FEBS Lett. 592, 2828–2844. 10.1002/1873-3468.13205 30058219 PMC6986807

[B50] Machnicka M. A. Olchowik A. Grosjean H. Bujnicki J. M. (2014). Distribution and frequencies of post-transcriptional modifications in tRNAs. RNA Biol. 11, 1619–1629. 10.4161/15476286.2014.992273 25611331 PMC4615829

[B51] Marck C. Grosjean H. (2003). Identification of BHB splicing motifs in intron-containing tRNAs from 18 archaea: evolutionary implications. RNA 9, 1516–1531. 10.1261/rna.5132503 14624007 PMC1370505

[B52] Mee Y. P. Wu G. Gonzalez-Sulser A. Vaucheret H. Poethig R. S. (2005). Nuclear processing and export of microRNAs in *Arabidopsis* . Proc. Natl. Acad. Sci. U. S. A. 102, 3691–3696. 10.1073/pnas.0405570102 15738428 PMC553294

[B53] Melton D. A. De Robertis E. M. Cortese R. (1980). Order and intracellular location of the events involved in the maturation of a spliced tRNA. Nature 284, 143–148. 10.1038/284143a0 6987526

[B54] Mohanty B. K. Kushner S. R. (2019). New insights into the relationship between tRNA processing and polyadenylation in *Escherichia coli* . Trends Genet. 35, 434–445. 10.1016/j.tig.2019.03.003 31036345 PMC7368558

[B55] Mort M. Ivanov D. Cooper D. N. Chuzhanova N. A. (2008). A meta-analysis of nonsense mutations causing human genetic disease. Hum. Mutat. 29, 1037–1047. 10.1002/humu.20763 18454449

[B56] Motorin Y. Grosjean H. (1999). Multisite-specific tRNA:m^5^C-methyltransferase (Trm4) in yeast *Saccharomyces cerevisiae*: identification of the gene and substrate specificity of the enzyme. RNA 5, 1105–1118. 10.1017/S1355838299982201 10445884 PMC1369833

[B57] Motorin Y. Keith G. Simon C. Foiret D. Simos G. Hurt E. (1998). The yeast tRNA:pseudouridine synthase Pus1p displays a multisite substrate specificity. RNA 4, 856–869. 10.1017/S1355838298980396 9671058 PMC1369665

[B58] Motorin Y. Lyko F. Helm M. (2009). 5-methylcytosine in RNA: detection, enzymatic formation and biological functions. Nucleic Acids Res. 38, 1415–1430. 10.1093/nar/gkp1117 20007150 PMC2836557

[B59] Müller M. Samel-Pommerencke A. Legrand C. Tuorto F. Lyko F. Ehrenhofer-Murray A. E. (2019). Division of labour: tRNA methylation by the NSun2 tRNA methyltransferases Trm4a and Trm4b in fission yeast. RNA Biol. 16, 249–256. 10.1080/15476286.2019.1568819 30646830 PMC6380293

[B60] Noma A. Kirino Y. Ikeuchi Y. Suzuki T. (2006). Biosynthesis of wybutosine, a hyper-modified nucleoside in eukaryotic phenylalanine tRNA. EMBO J. 25, 2142–2154. 10.1038/sj.emboj.7601105 16642040 PMC1462984

[B61] Ohira T. Suzuki T. (2011). Retrograde nuclear import of tRNA precursors is required for modified base biogenesis in yeast. Proc. Natl. Acad. Sci. U. S. A. 108, 10502–10507. 10.1073/pnas.1105645108 21670254 PMC3127885

[B62] Paushkin S. V. Patel M. Furia B. S. Peltz S. W. Trotta C. R. (2004). Identification of a human endonuclease complex reveals a link between tRNA splicing and pre-mRNA 3′ end formation. Cell 117, 311–321. 10.1016/S0092-8674(04)00342-3 15109492

[B63] Perche-Letuvée P. Molle T. Forouhar F. Mulliez E. Atta M. (2014). Wybutosine biosynthesis: structural and mechanistic overview. RNA Biol. 11, 1508–1518. 10.4161/15476286.2014.992271 25629788 PMC4615248

[B64] Phizicky E. M. Hopper A. K. (2023). The life and times of a tRNA. RNA 29, 898–957. 10.1261/rna.079620.123 37055150 PMC10275265

[B65] Popow J. Schleiffer A. Martinez J. (2012). Diversity and roles of (t)RNA ligases. Cell. Mol. Life Sci. 69, 2657–2670. 10.1007/s00018-012-0944-2 22426497 PMC3400036

[B66] Purchal M. K. Eyler D. E. Tardu M. Franco M. K. Korn M. M. Khan T. (2022). Pseudouridine synthase 7 is an opportunistic enzyme that binds and modifies substrates with diverse sequences and structures. Proc. Natl. Acad. Sci. U. S. A. 119, e2109708119. 10.1073/pnas.2109708119 35058356 PMC8794802

[B67] Ramos J. Fu D. (2019). The emerging impact of tRNA modifications in the brain and nervous system. Biochim. Biophys. Acta - Gene Regul. Mech. 1862, 412–428. 10.1016/j.bbagrm.2018.11.007 30529455

[B68] Reinhold-Hurek B. Shub D. A. (1992). Self-splicing introns in tRNA genes of widely divergent bacteria. Nature 357, 173–176. 10.1038/357173a0 1579169

[B69] Rintala-Dempsey A. C. Kothe U. (2017). Eukaryotic stand-alone pseudouridine synthases–RNA modifying enzymes and emerging regulators of gene expression? RNA Biol. 14, 1185–1196. 10.1080/15476286.2016.1276150 28045575 PMC5699540

[B70] Rubio M. A. T. Paris Z. Gaston K. W. Fleming I. M. C. Sample P. Trotta C. R. (2013). Unusual noncanonical intron editing is important for tRNA splicing in *Trypanosoma brucei* . Mol. Cell 52, 184–192. 10.1016/j.molcel.2013.08.042 24095278 PMC3825838

[B71] Schimmel P. (2018). The emerging complexity of the tRNA world: mammalian tRNAs beyond protein synthesis. Nat. Rev. Mol. Cell Biol. 19, 45–58. 10.1038/nrm.2017.77 28875994

[B72] Schwartz S. Bernstein D. A. Mumbach M. R. Jovanovic M. Herbst R. H. León-Ricardo B. X. (2014). Transcriptome-wide mapping reveals widespread dynamic-regulated pseudouridylation of ncRNA and mRNA. Cell 159, 148–162. 10.1016/j.cell.2014.08.028 25219674 PMC4180118

[B73] Sekulovski S. Trowitzsch S. (2022). Transfer RNA processing-from a structural and disease perspective. Biol. Chem. 403, 749–763. 10.1515/hsz-2021-0406 35728022

[B74] Simos G. Tekotte H. Grosjean H. Segref A. Sharma K. Tollervey D. (1996). Nuclear pore proteins are involved in the biogenesis of functional tRNA. EMBO J. 15, 2270–2284. 10.1002/j.1460-2075.1996.tb00580.x 8641292 PMC450152

[B75] Singh S. K. Gurha P. Tran E. J. Maxwell E. S. Gupta R. (2004). Sequential 2′-O-methylation of archaeal pre-tRNA^Trp^ nucleotides is guided by the intron-encoded but *trans*-acting box C/D ribonucleoprotein of pre-tRNA. J. Biol. Chem. 279, 47661–47671. 10.1074/jbc.M408868200 15347671

[B76] Spenkuch F. Motorin Y. Helm M. (2014). Pseudouridine: still mysterious, but never a fake (uridine). RNA Biol. 11, 1540–1554. 10.4161/15476286.2014.992278 25616362 PMC4615568

[B77] Strobel M. C. Abelson J. (1986). Effect of intron mutations on processing and function of *Saccharomyces cerevisiae SUP53* tRNA *in vitro* and *in vivo* . Mol. Cell. Biol. 6, 2663–2673. 10.1128/mcb.6.7.2663 3537724 PMC367823

[B78] Sugahara J. Yachie N. Arakawa K. Tomita M. (2007). *In silico* screening of archaeal tRNA-encoding genes having multiple introns with bulge-helix-bulge splicing motifs. RNA 13, 671–681. 10.1261/rna.309507 17369313 PMC1852824

[B79] Suzuki T. (2021). The expanding world of tRNA modifications and their disease relevance. Nat. Rev. Mol. Cell Biol. 22, 375–392. 10.1038/s41580-021-00342-0 33658722

[B80] Szweykowska-Kulinska Z. Beier H. (1992). Sequence and structure requirements for the biosynthesis of pseudouridine (Ψ_35_) in plant pre-tRNA^Tyr^ . EMBO J. 11, 1907–1912. 10.1002/j.1460-2075.1992.tb05243.x 1582418 PMC556649

[B81] Szweykowska-Kulinska Z. Senger B. Keith G. Fasiolo F. Grosjean H. (1994). Intron-dependent formation of pseudouridines in the anticodon of *Saccharomyces cerevisiae* minor tRNA^Ile^ . EMBO J. 13, 4636–4644. 10.1002/j.1460-2075.1994.tb06786.x 7925304 PMC395397

[B82] Tanner M. Cech T. (1996). Activity and thermostability of the small self-splicing group I intron in the pre-tRNA^lle^ of the purple bacterium *Azoarcus* . RNA 2, 74–83. Available at: http://www.ncbi.nlm.nih.gov/pubmed/1369352. 8846298 PMC1369352

[B83] Thiebe R. Zachau H. G. (1968). A specific modification next to the anticodon of phenylalanine transfer ribonucleic acid. Eur. J. Biochem. 5, 546–555. 10.1111/j.1432-1033.1968.tb00404.x 5698615

[B84] Tocchini-Valentini G. D. Fruscoloni P. Tocchini-Valentini G. P. (2005). Coevolution of tRNA intron motifs and tRNA endonuclease architecture in Archaea. Proc. Natl. Acad. Sci. U. S. A. 102, 15418–15422. 10.1073/pnas.0506750102 16221764 PMC1266117

[B85] Tocchini-Valentini G. D. Fruscoloni P. Tocchini-Valentini G. P. (2009). Processing of multiple-intron-containing pretRNA. Proc. Natl. Acad. Sci. U. S. A. 106, 20246–20251. 10.1073/pnas.0911658106 19910528 PMC2787110

[B86] Tocchini-Valentini G. D. Fruscoloni P. Tocchini-Valentini G. P. (2011). Evolution of introns in the archaeal world. Proc. Natl. Acad. Sci. U. S. A. 108, 4782–4787. 10.1073/pnas.1100862108 21383132 PMC3064391

[B87] Tocchini-Valentini G. D. Tocchini-Valentini G. P. (2021). Archaeal tRNA-splicing endonuclease as an effector for RNA recombination and novel trans-splicing pathways in eukaryotes. J. Fungi 7, 1069. 10.3390/jof7121069 PMC8707768 34947051

[B88] Trotta C. R. Miao F. Arn E. A. Stevens S. W. Ho C. K. Rauhut R. (1997). The yeast tRNA splicing endonuclease: a tetrameric enzyme with two active site subunits homologous to the archaeal tRNA endonucleases. Cell 89, 849–858. 10.1016/S0092-8674(00)80270-6 9200603

[B89] Urban A. Behm-Ansmant I. Branlant C. Motorlin Y. (2009). RNA sequence and two-dimensional structure features required for efficient substrate modification by the *Saccharomyces cerevisiae* RNA:Ψ-synthase Pus7p. J. Biol. Chem. 284, 5845–5858. 10.1074/jbc.M807986200 19114708

[B90] van Tol H. Beier H. (1988). All human tRNA^Tyr^ genes contain introns as a prerequisite for pseudouridine biosynthesis in the anticodon. Nucleic Acids Res. 16, 1951–1966. 10.1093/nar/16.5.1951 3357766 PMC338192

[B91] Waas W. F. Druzina Z. Hanan M. Schimmel P. (2007). Role of a tRNA base modification and its precursors in frameshifting in eukaryotes. J. Biol. Chem. 282, 26026–26034. 10.1074/jbc.M703391200 17623669

[B92] Wang C. Jia Q. Zeng J. Chen R. Xie W. (2017). Structural insight into the methyltransfer mechanism of the bifunctional Trm5. Sci. Adv. 3, e1700195. 10.1126/sciadv.1700195 29214216 PMC5714064

[B93] Wang Z. Xu X. Li X. Fang J. Huang Z. Zhang M. (2023). Investigations of single-subunit tRNA methyltransferases from yeast. J. Fungi 9, 1030. 10.3390/jof9101030 PMC10608323 37888286

[B94] Watkins N. J. Bohnsack M. T. (2012). The box C/D and H/ACA snoRNPs: key players in the modification, processing and the dynamic folding of ribosomal RNA. WIREs RNA 3, 397–414. 10.1002/wrna.117 22065625

[B95] Yoshihisa T. (2014). Handling tRNA introns, archaeal way and eukaryotic way. Front. Genet. 5, 213. 10.3389/fgene.2014.00213 25071838 PMC4090602

[B96] Yoshihisa T. Ohshima C. Yunoki-Esaki K. Endo T. (2007). Cytoplasmic splicing of tRNA in *Saccharomyces cerevisiae* . Genes Cells 12, 285–297. 10.1111/j.1365-2443.2007.01056.x 17352735

[B97] Yoshihisa T. Yunoki-Esaki K. Ohshima C. Tanaka N. Endo T. (2003). Possibility of cytoplasmic pre-tRNA splicing: the yeast tRNA splicing endonuclease mainly localizes on the mitochondria. Mol. Biol. Cell 14, 3266–3279. 10.1091/mbc.e02-11-0757 12925762 PMC181566

[B98] Zerfass K. Beier H. (1992). Pseudouridine in the anticodon GΨA of plant cytoplasmic tRNA^Tyr^ is required for UAG and UAA suppression in the TMV-specific context. Nucleic Acids Res. 20, 5911–5918. 10.1093/nar/20.22.5911 1461724 PMC334454

[B99] Zhang W. Foo M. Eren A. M. Pan T. (2022). tRNA modification dynamics from individual organisms to metaepitranscriptomics of microbiomes. Mol. Cell 82, 891–906. 10.1016/j.molcel.2021.12.007 35032425 PMC8897278

[B100] Zhang X. Yang F. Zhan X. Bian T. Xing Z. Lu Y. (2023). Structural basis of pre-tRNA intron removal by human tRNA splicing endonuclease. Mol. Cell 83, 1328–1339.e4. 10.1016/j.molcel.2023.03.015 37028420

